# Supplementary material for: AffyMAPSDetector: a software tool to characterize Affymetrix GeneChip™ expression arrays with respect to SNPs
Source: BMC Bioinformatics. 2007 Jul 30;8:276. doi: 10.1186/1471-2105-8-276 (PMC1959249; doi:10.1186/1471-2105-8-276)
Supplement: Additional file 13 — AffyMAPSDetector v1 source code. AffyMAPSDetector source code. [file 1471-2105-8-276-S13.zip › AffyMAPSDetector_v1_src/doc/serialized-form.html]

Serialized Form


|  |  |  |  |
| --- | --- | --- | --- |
| **Overview** \| Package \| Class \| Use \| **Tree** \| **Deprecated** \| **Index** \| **Help** | | |  |
| PREV   NEXT | **FRAMES**    **NO FRAMES**     **All Classes** |  |  |


---


# Serialized Form


---

**Package** **affychip.snpdetector.model**

**Class affychip.snpdetector.model.DataState extends java.lang.Object implements Serializable**

**Serialization Methods**

### readObject

```
private void readObject(java.io.ObjectInputStream ois)
                 throws java.lang.ClassNotFoundException,
                        java.io.IOException
```

---


### writeObject

```
private void writeObject(java.io.ObjectOutputStream oos)
                  throws java.io.IOException
```

**Serialized Fields**

### geneInfoFile

```
java.io.File geneInfoFile
```

---

### probeInfoFile

```
java.io.File probeInfoFile
```

---

### geneInfoFieldSeparator

```
java.lang.String geneInfoFieldSeparator
```

---

### probeInfoFieldSeparator

```
java.lang.String probeInfoFieldSeparator
```

---

### geneInfoHeaders

```
java.lang.String[] geneInfoHeaders
```

---

### probeInfoHeaders

```
java.lang.String[] probeInfoHeaders
```

---

### geneInfoMap

```
java.util.HashMap geneInfoMap
```

---

### probeInfoMap

```
java.util.HashMap probeInfoMap
```

---

### geneInfo

```
GeneInfo[] geneInfo
```

---

### probeSet

```
java.util.HashMap probeSet
```

---

### geneIdentifierFieldIndex

```
int geneIdentifierFieldIndex
```

---

### locusLinkFieldIndex

```
int locusLinkFieldIndex
```

---

### geneInfoProbeSetIdFieldIndex

```
int geneInfoProbeSetIdFieldIndex
```

---

### probeInfoProbeSetIdFieldIndex

```
int probeInfoProbeSetIdFieldIndex
```

---

### xPosFieldIndex

```
int xPosFieldIndex
```

---

### yPosFieldIndex

```
int yPosFieldIndex
```

---

### mmPosFieldIndex

```
int mmPosFieldIndex
```

---

### probeSeqFieldIndex

```
int probeSeqFieldIndex
```

---

### noLocusLinkFilename

```
java.lang.String noLocusLinkFilename
```

---

### hasSnpsFilename

```
java.lang.String hasSnpsFilename
```

---

### noSnpsFilename

```
java.lang.String noSnpsFilename
```

---

### webGeneInfoFilename

```
java.lang.String webGeneInfoFilename
```

---

### webSnpInfoFilename

```
java.lang.String webSnpInfoFilename
```

---

### organism

```
java.lang.String organism
```

---

**Package** **jwf**

**Class jwf.ErrorMessageBox extends javax.swing.JDialog implements Serializable**

**Serialized Fields**

### textPane

```
javax.swing.JTextPane textPane
```

**Class jwf.NullWizardPanel extends WizardPanel implements Serializable**

**Class jwf.Wizard extends javax.swing.JPanel implements Serializable**

**Serialized Fields**

### backButton

```
javax.swing.JButton backButton
```

---

### nextButton

```
javax.swing.JButton nextButton
```

---

### finishButton

```
javax.swing.JButton finishButton
```

---

### cancelButton

```
javax.swing.JButton cancelButton
```

---

### helpButton

```
javax.swing.JButton helpButton
```

---

### listeners

```
java.util.HashMap listeners
```

---

### previous

```
java.util.Stack previous
```

---

### current

```
WizardPanel current
```

---

### ctx

```
WizardContext ctx
```

---

### i18n

```
java.util.Map i18n
```

**Class jwf.WizardPanel extends javax.swing.JPanel implements Serializable**

**Serialized Fields**

### wizardContext

```
WizardContext wizardContext
```

:   The context of the wizard process.

---

**Package** **affychip.snpdetector.util**

**Class affychip.snpdetector.util.ComplementDnaSeq extends javax.swing.JFrame implements Serializable**

**Serialized Fields**

### inputLabel

```
javax.swing.JLabel inputLabel
```

---

### inputSeqField

```
javax.swing.JTextField inputSeqField
```

---

### complementarySeqTextArea

```
javax.swing.JTextArea complementarySeqTextArea
```

---

### actionBtn

```
javax.swing.JButton actionBtn
```

**Class affychip.snpdetector.util.DuplicateRemover extends javax.swing.JFrame implements Serializable**

**Serialized Fields**

### fileChooser

```
javax.swing.JFileChooser fileChooser
```

---

### file

```
java.io.File file
```

**Class affychip.snpdetector.util.GeneAnnotLoaderWizard extends javax.swing.JDialog implements Serializable**

**Serialized Fields**

### logger

```
org.apache.log4j.Logger logger
```

---

### wizStep

```
GeneAnnotLoaderWizard.GeneAnnotFileLoaderStepPanel wizStep
```

---

### heatmapModel

```
PmMmHeatmapViewerModel heatmapModel
```

---

### parent

```
java.awt.Frame parent
```

**Class affychip.snpdetector.util.PmMmHeatMapViewer extends javax.swing.JFrame implements Serializable**

**Serialized Fields**

### curView

```
java.lang.String curView
```

---

### lineStr

```
java.lang.String lineStr
```

---

### removedRows

```
java.util.Vector removedRows
```

---

### selectedRow

```
int selectedRow
```

---

### selectedCol

```
int selectedCol
```

---

### replicateMap

```
java.util.HashMap replicateMap
```

---

### heatmapModel

```
PmMmHeatmapViewerModel heatmapModel
```

---

### table

```
javax.swing.JTable table
```

---

### popup

```
javax.swing.JPopupMenu popup
```

---

### removeRowsMi

```
javax.swing.JMenuItem removeRowsMi
```

---

### conflictingSnpCallsMi

```
javax.swing.JMenuItem conflictingSnpCallsMi
```

---

### restoreCellMi

```
javax.swing.JMenuItem restoreCellMi
```

---

### restoreRowMi

```
javax.swing.JMenuItem restoreRowMi
```

---

### restoreAllMi

```
javax.swing.JMenuItem restoreAllMi
```

---

### splitPane

```
javax.swing.JSplitPane splitPane
```

---

### model

```
PmMmHeatMapViewer.HeatmapTableModel model
```

---

### tabs

```
javax.swing.JTabbedPane tabs
```

---

### menuBar

```
javax.swing.JMenuBar menuBar
```

---

### fileMenu

```
javax.swing.JMenu fileMenu
```

---

### openMI

```
javax.swing.JMenuItem openMI
```

---

### saveAsMI

```
javax.swing.JMenuItem saveAsMI
```

---

### exitMI

```
javax.swing.JMenuItem exitMI
```

---

### viewMenu

```
javax.swing.JMenu viewMenu
```

---

### toggleViewMI

```
javax.swing.JMenuItem toggleViewMI
```

---

### viewRemovedRowsMI

```
javax.swing.JMenuItem viewRemovedRowsMI
```

---

### findGeneMI

```
javax.swing.JMenuItem findGeneMI
```

---

### addGeneAnnotMI

```
javax.swing.JMenuItem addGeneAnnotMI
```

---

### analysisMenu

```
javax.swing.JMenu analysisMenu
```

---

### fullGenotypeAnalysisMI

```
javax.swing.JMenuItem fullGenotypeAnalysisMI
```

---

### locusGenotypeAnalysisMI

```
javax.swing.JMenuItem locusGenotypeAnalysisMI
```

---

### uniqueSnpSignatureMI

```
javax.swing.JMenuItem uniqueSnpSignatureMI
```

---

### toolBar

```
javax.swing.JToolBar toolBar
```

---

### geneList

```
javax.swing.JList geneList
```

---

### clusterArea

```
javax.swing.JTextPane clusterArea
```

---

### basic

```
javax.swing.text.Style basic
```

---

### regular

```
javax.swing.text.Style regular
```

---

### line

```
javax.swing.text.Style line
```

---

### heading

```
javax.swing.text.Style heading
```

---

### pmMmEqParamTF

```
javax.swing.JTextField pmMmEqParamTF
```

---

### pmMmEqParamBtn

```
javax.swing.JButton pmMmEqParamBtn
```

---

### filterBtn

```
javax.swing.JButton filterBtn
```

---

### slider

```
javax.swing.JSlider slider
```

---

### pmMmCutOffRange

```
javax.swing.JLabel pmMmCutOffRange
```

---

### conflictingSnpCalls

```
javax.swing.JLabel conflictingSnpCalls
```

---

### missedSnpCalls

```
int missedSnpCalls
```

---

**Package** **affychip.snpdetector.model.event**

**Class affychip.snpdetector.model.event.StatusEvent extends java.util.EventObject implements Serializable**

**Serialized Fields**

### message

```
java.lang.String message
```

---

### visualArtifact

```
java.awt.Color visualArtifact
```

---

**Package** **affychip.snpdetector.ui**

**Class affychip.snpdetector.ui.AffyMapsDetectorFrame extends javax.swing.JInternalFrame implements Serializable**

**Serialized Fields**

### parent

```
javax.swing.JFrame parent
```

---

### menubar

```
javax.swing.JMenuBar menubar
```

---

### toolbar

```
javax.swing.JToolBar toolbar
```

---

### fileMenu

```
javax.swing.JMenu fileMenu
```

---

### viewMenu

```
javax.swing.JMenu viewMenu
```

---

### viewGeneInfoMenuItem

```
javax.swing.JMenuItem viewGeneInfoMenuItem
```

---

### viewProbeInfoMenuItem

```
javax.swing.JMenuItem viewProbeInfoMenuItem
```

---

### startMenuItem

```
javax.swing.JMenuItem startMenuItem
```

---

### stopMenuItem

```
javax.swing.JMenuItem stopMenuItem
```

---

### table

```
javax.swing.JTable table
```

---

### tableScroller

```
javax.swing.JScrollPane tableScroller
```

---

### colNames

```
java.lang.String[] colNames
```

---

### colClasses

```
java.lang.Class[] colClasses
```

---

### tableData

```
java.util.Vector tableData
```

---

### statusBar

```
javax.swing.JTextField statusBar
```

---

### statusBarSplitPane

```
javax.swing.JSplitPane statusBarSplitPane
```

---

### progressBar

```
javax.swing.JProgressBar progressBar
```

---

### ds

```
DataState ds
```

---

### snpMatcher

```
SnpMatcher snpMatcher
```

**Class affychip.snpdetector.ui.AffyMapsDetectorUI extends javax.swing.JFrame implements Serializable**

**Serialized Fields**

### desktop

```
javax.swing.JDesktopPane desktop
```

---

### affyMapsDetectorFrame

```
AffyMapsDetectorFrame affyMapsDetectorFrame
```

---

### crossHbCheckFrame

```
CrossHbCheckFrame crossHbCheckFrame
```

---

### menubar

```
javax.swing.JMenuBar menubar
```

---

### toolbar

```
javax.swing.JToolBar toolbar
```

---

### toolsMenu

```
javax.swing.JMenu toolsMenu
```

---

### helpMenu

```
javax.swing.JMenu helpMenu
```

---

### windowsMenu

```
javax.swing.JMenu windowsMenu
```

---

### memoryBar

```
javax.swing.JProgressBar memoryBar
```

**Class affychip.snpdetector.ui.CrossHbCheckFrame extends javax.swing.JInternalFrame implements Serializable**

**Serialized Fields**

### parent

```
javax.swing.JFrame parent
```

**Class affychip.snpdetector.ui.FileLoaderWizardDialog extends javax.swing.JDialog implements Serializable**

**Serialized Fields**

### logger

```
org.apache.log4j.Logger logger
```

---

### parent

```
AffyMapsDetectorUI parent
```

---

### wizStep

```
StepOnePanel wizStep
```

**Class affychip.snpdetector.ui.StepOnePanel extends WizardPanel implements Serializable**

**Serialized Fields**

### geneInfoFieldSeparatorCB

```
javax.swing.JComboBox geneInfoFieldSeparatorCB
```

---

### logger

```
org.apache.log4j.Logger logger
```

---

### organismsCB

```
javax.swing.JComboBox organismsCB
```

---

### geneIdentifierCB

```
javax.swing.JComboBox geneIdentifierCB
```

---

### locusLinkCB

```
javax.swing.JComboBox locusLinkCB
```

---

### probeSetIdCB

```
javax.swing.JComboBox probeSetIdCB
```

---

### geneIdentifierLB

```
javax.swing.JLabel geneIdentifierLB
```

---

### locusLinkLB

```
javax.swing.JLabel locusLinkLB
```

---

### probeSetIdLB

```
javax.swing.JLabel probeSetIdLB
```

---

### nextStep

```
StepTwoPanel nextStep
```

**Class affychip.snpdetector.ui.StepThreePanel extends WizardPanel implements Serializable**

**Serialized Fields**

### noLocusLinkTF

```
javax.swing.JTextField noLocusLinkTF
```

---

### hasSnpsTF

```
javax.swing.JTextField hasSnpsTF
```

---

### noSnpsTF

```
javax.swing.JTextField noSnpsTF
```

---

### webGeneInfoTF

```
javax.swing.JTextField webGeneInfoTF
```

---

### webSnpInfoTF

```
javax.swing.JTextField webSnpInfoTF
```

---

### noLocusLinkLB

```
javax.swing.JLabel noLocusLinkLB
```

---

### hasSnpsLB

```
javax.swing.JLabel hasSnpsLB
```

---

### noSnpsLB

```
javax.swing.JLabel noSnpsLB
```

---

### webGeneInfoLB

```
javax.swing.JLabel webGeneInfoLB
```

---

### webSnpInfoLB

```
javax.swing.JLabel webSnpInfoLB
```

---

### noLocusLinkFilename

```
java.lang.String noLocusLinkFilename
```

---

### hasSnpsFilename

```
java.lang.String hasSnpsFilename
```

---

### noSnpsFilename

```
java.lang.String noSnpsFilename
```

---

### webGeneInfoFilename

```
java.lang.String webGeneInfoFilename
```

---

### webSnpInfoFilename

```
java.lang.String webSnpInfoFilename
```

**Class affychip.snpdetector.ui.StepTwoPanel extends WizardPanel implements Serializable**

**Serialized Fields**

### probeSetIdCB

```
javax.swing.JComboBox probeSetIdCB
```

---

### xPosCB

```
javax.swing.JComboBox xPosCB
```

---

### yPosCB

```
javax.swing.JComboBox yPosCB
```

---

### mmPosCB

```
javax.swing.JComboBox mmPosCB
```

---

### probeSeqCB

```
javax.swing.JComboBox probeSeqCB
```

---

### probeSetIdLB

```
javax.swing.JLabel probeSetIdLB
```

---

### xPosLB

```
javax.swing.JLabel xPosLB
```

---

### yPosLB

```
javax.swing.JLabel yPosLB
```

---

### mmPosLB

```
javax.swing.JLabel mmPosLB
```

---

### probeSeqLB

```
javax.swing.JLabel probeSeqLB
```

---

### probeInfoFieldSeparatorCB

```
javax.swing.JComboBox probeInfoFieldSeparatorCB
```

---

### logger

```
org.apache.log4j.Logger logger
```

---

### nextStep

```
StepThreePanel nextStep
```

---


|  |  |  |  |
| --- | --- | --- | --- |
| **Overview** \| Package \| Class \| Use \| **Tree** \| **Deprecated** \| **Index** \| **Help** | | |  |
| PREV   NEXT | **FRAMES**    **NO FRAMES**     **All Classes** |  |  |


---
